# Supplementary material for: HistoPerm: A permutation-based view generation approach for improving histopathologic feature representation learning
Source: J Pathol Inform. 2023 Jul 4;14:100320. doi: 10.1016/j.jpi.2023.100320 (PMC10339175; doi:10.1016/j.jpi.2023.100320)
Supplement: Supplementary file 1 — Supplementary material [file mmc1.docx]

Supplementary Material

Appendix A. Hyperparameter Search on $\boldsymbol{\alpha}$

**Supplementary Figure 1.** Patch-level linear evaluation mode results over $\alpha$, the percentage of the mini-batch eligible for permutation. We report all results on the respective development sets.

To determine the optimal value of $\alpha$, we perform a search over $\alpha\in${0, 0.25, 0.5, 0.75, 1}. Of note, $\alpha=$0 is equivalent to the standard BYOL, SimCLR, and VICReg methods, as all elements of the mini-batch are considered unlabeled. We present our results in Supplementary Figure 1 for the patch-level linear evaluation mode on the respective development sets. From our experiments over $\alpha$, we conclude that selecting $\alpha=$0.75 is a good choice for both datasets, as in all cases, it outperforms the standard methods in terms of accuracy. We find that almost any value of $\alpha$ results in improved performance over the respective baseline methods in terms of accuracy, indicating that most permutation choices should provide a measurable improvement. Lastly, even suboptimal values of $\alpha$produce promising results, so expensive tuning of this hyperparameter may not be necessary in most cases.

Appendix B. Data Transformation Hyperparameter Search

We performed a hyperparameter search on the applied image augmentations for use in the pretraining phase. The tested image augmentations are as follows:

- **Cropping:** Randomly sample a crop from the image between 8% and 100% of the original image size (224$\times$224 pixels for our datasets). Additionally, randomly sample an aspect ratio between 3/4 and 4/3. Resize images to 224$\times$224 pixels using bilinear interpolation.
- **Flipping:** Flip the images over the horizontal and vertical axes. Flip over both axes, as histology images are rotation invariant.
- **Color Jittering:** Randomly change the brightness, contrast, hue, and saturation of the image according to a value uniformly selected from a range.
- **Grayscale:** Convert the image to grayscale. Given intensities $\left( r,g,b \right)$ for a pixel in the image, convert it to grayscale according to the formula: $0.299r+0.587g+0.114b$.
- **Gaussian Blurring:** Use a 23$\times$23-pixel Gaussian kernel to apply blurring with the standard deviation sampled from the range [0.1, 2.0].
- **Solarization:** Invert all pixels in the image above a threshold.

We use the Kornia computer vision deep learning library for all augmentations^1^. In Supplementary Table 1, we provide the hyperparameters for the tested data transformations.

| Hyperparameter | $\mathcal{T}_{\boldsymbol{1}}$ | $\mathcal{T}_{\boldsymbol{2}}$ |
| --- | --- | --- |
| Cropping probability | 1.0 | 1.0 |
| Flipping probability | 0.5 | 0.5 |
| Color jittering  probability  brightness factor  contrast factor  hue factor  saturation factor | 0.8  0.4  0.4  0.1  0.2 | 0.8  0.4  0.4  0.1  0.2 |
| Grayscale probability | 0.2 | 0.2 |
| Gaussian blurring probability | 1.0 | 0.1 |
| Solarization  probability  threshold | 0.0  128/255 | 0.2  128/255 |

**Supplementary Table 1.** Hyperparameter settings for tested data transformations.

## **Appendix B.1 Data Transformation Set**

We detail the tested data transformation sets as follows:

- **Base:** Apply all transformations as enumerated in Supplementary Table 1.
- **Remove Grayscale:** Apply all transformations as enumerated in Supplementary Table 1, apart from random conversion to grayscale.
- **Remove Color:** Apply all transformations as enumerated in Supplementary Table 1, apart from random conversion to grayscale, color jittering, and solarization.
- **Crop + Blur + Flipping:** Apply cropping, Gaussian blurring, and flipping as enumerated in Supplementary Table 1.
- **Crop + Flipping:** Apply cropping and flipping as enumerated in Supplementary Table 1.
- **Blur + Flipping:** Apply Gaussian blurring and flipping as enumerated in Supplementary Table 1.

**Supplementary Figure 2.** Linear performance results on the CD and RCC development sets for the tested data transformations.

In Supplementary Figure 2, we present the results of our hyperparameter search on the data transformations. Overall, we find that adding HistoPerm provides a measurable classification accuracy improvement in all cases apart from “Crop + Flipping” on the CD dataset. We select “Crop + Blur + Flipping” as the best performing set of data transformations, because it performs well both with and without HistoPerm and should provide the most equitable base for demonstrating the benefits of our approach. Using only “Blur + Flipping” does result in overall higher classification accuracy with HistoPerm, except the results without HistoPerm are significantly lower and are not representative of a baseline model.

Appendix C. Architecture Details

In a joint embedding architecture, there are two or three main components depending on the method being used. BYOL consists of an encoder, projector, and predictor, SimCLR uses an encoder and projector, and VICReg uses an encoder and expander. We discuss the structure of each component below.

1. **Encoder:** ResNet18^2^ feature extractor (i.e., the output of the final average pooling layer), $f$, producing representation $y$ such that $y=f\left( x \right)\in\mathbb{R}^{\mathcal{D}_{f}}$. We use $\mathcal{D}_{f}=$512 in all experiments.
2. **Projector:** Multilayer perceptron, $g$, mapping $y$to $z=g\left( y \right)\in\mathbb{R}^{\mathcal{D}_{g}}$. We instantiate $g$ as a two-layer multilayer perceptron with a single hidden layer of size 4096 and output size $\mathcal{D}_{g}=$ 256.
3. **Expander:** Multilayer perceptron, $h$, mapping $y$to $z=h\left( y \right)\in\mathbb{R}^{\mathcal{D}_{h}}$*.* We instantiate $h$as a two-layer multilayer perceptron with a single hidden layer of size 2048 and output size $\mathcal{D}_{h}=$ 2048.
4. **Predictor:** Multilayer perceptron, $q$, mapping $z$ to $p=q\left( z \right)\in\mathbb{R}^{\mathcal{D}_{q}}$. We instantiate $q$ as a two-layer multilayer perceptron with a single hidden layer of size 4096 and output size $\mathcal{D}_{q}=$ 256.

Now, we cover how these components are used in the standard BYOL, SimCLR, and VICReg methods as well as when HistoPerm is added.

## **Appendix C.1 BYOL**

The BYOL architecture is split across two components called online and target branches, parameterized by $\theta$ and $\xi$, respectively. The online branch is composed of three stages: encoder $f_{\theta}$, projector $g_{\theta}$, and predictor $q_{\theta}$. Likewise, the target branch has two stages: encoder $f_{\xi}$ and projector $g_{\xi}$. Encoders $f_{\theta}$ and $f_{\xi}$ map input views to a representation space, which are then fed to respective projectors $g_{\theta}$ and $g_{\xi}$. Note that the predictor $q_{\theta}$ is only used in the online network, as prior works have shown that this architectural asymmetry is necessary to avoid collapsing to the trivial solution.^3^ At the end of training, we only keep the online encoder $f_{\theta}$ and use the pretrained weights as initialization for the fully-supervised downstream tasks.

Learning progresses by computing the mean squared error between online and target branches. For both unlabeled and labeled views, we compute the loss as follows:

$$\mathcal{L}_{BYOL}=\underset{{Loss}_{u}}{\underbrace{\left\| q_{\theta}(z_{u,1})-\mathrm{sg}(z_{u,2}) \right\|}}+\underset{{Loss}_{l}}{\underbrace{\left\| q_{\theta}(z_{l,1})-\mathrm{sg}(\tilde{z}_{l,2}) \right\|}}$$

where sg($\cdot$) is the stop-gradient operation, so the target branch weights are not updated through optimization. As shown in the SimSiam paper^3^, the stop-gradient is necessary to avoid collapse. We symmetrize the loss by passing $v_{u,2}$, $\tilde{v}_{l, 2}$ and $v_{u,1}$, $v_{l,1}$ to the online and target branches, respectively. In the code implementation for the loss function, we combine and process both mini-batches simultaneously to avoid inadvertently leaking any information about the source dataset through batch normalization. Note that in the case where $v_{l,1}=\tilde{v}_{l, 2} =\emptyset,$(i.e., $\mathcal{P}_{l}=\mathcal{P}_{u}=\emptyset$)*,* our method reduces to the default BYOL formulation.

Then, we perform the weight updates as follows:

$$\theta\leftarrow\mathrm{optimizer}(\theta, \nabla_{\theta}\mathcal{L,}\eta)$$

$$\xi\leftarrow\tau\xi+\left( 1-\tau\right)\xi$$

where $\tau\in$ [0, 1] is a momentum hyperparameter and $\eta$ is the learning rate for gradient descent.

## **Appendix C.2 SimCLR**

The SimCLR architecture consists of an encoder $f_{\phi}$ and projector $g_{\phi}$ parameterized by weights $\phi$. The encoder $f_{\phi}$ maps pairs of input views to representations that are then fed to the projector $g_{\phi}$. Finally, $L_{2}$-normalization is applied to the outputs of the projector. At the end of training, we only keep the encoder $f_{\phi}$ and use the pretrained weights as initialization for the fully-supervised downstream tasks.

Learning progresses using the NT-Xent loss as defined in the original SimCLR formulation.^4^ For both unlabeled and labeled views, we compute the loss as follows:

$$\mathcal{L}_{SimCLR}=\frac{1}{\left| \mathcal{X}_{u} \right|+\left| \mathcal{X}_{l} \right|}\left[ \underset{{Loss}_{u}}{\underbrace{\sum_{i=1}^{\left| \mathcal{X}_{u} \right|} -\log\frac{\exp\left( z_{i}\cdot z_{i}^{'}/\tau\right)}{\sum_{k=1}^{\left| \mathcal{X}_{u} \right|+\left| \mathcal{X}_{l} \right|} \exp\left( z_{i}\cdot z_{k}/\tau\right)}}}+\underset{{Loss}_{l}}{\underbrace{\sum_{i=\left| \mathcal{X}_{u} \right|+1}^{\left| \mathcal{X}_{u} \right|+\left| \mathcal{X}_{l} \right|} -\log\frac{\exp\left( z_{i}\cdot z_{i}^{'}/\tau\right)}{\sum_{k=1}^{\left| \mathcal{X}_{u} \right|+\left| \mathcal{X}_{l} \right|} \exp\left( z_{i}\cdot z_{k}/\tau\right)}}} \right]$$

where $\tau$ is the temperature hyperparameter. Note that in the case where $v_{l,1}=\tilde{v}_{l, 2} =\emptyset,$(i.e., $\mathcal{P}_{l}=\mathcal{P}_{u}=\emptyset$), our method reduces to the default SimCLR formulation.

Then, we perform the weight updates as follows:

$$\phi\leftarrow\text{optimizer}\left( \phi,\nabla_{\phi}\mathcal{L,}\eta\right)$$

where $\eta$ is the learning rate for gradient descent.

## **Appendix C.3 VICReg**

The VICReg architecture consists of an encoder $f_{\psi}$ and expander $h_{\psi}$ parametrized by weights $\psi$. The encoder $f_{\psi}$ maps pairs of input views to representations that are then fed to the expander $h_{\psi}$. At the end of training, we only keep the encoder $f_{\psi}$ and use the pretrained weights as initialization for the fully-supervised downstream tasks.

Learning progresses using variance, invariance, and covariance loss terms.^5^ The variance term is as follows:

$$v\left( Z \right)=\frac{1}{d}\sum_{j=1}^{d} \max\left( 0,\gamma-S\left( z^{j},\epsilon\right) \right)$$

where

$$S\left( x,\epsilon\right)=\sqrt{\mathrm{Var}\left( x \right)+\epsilon}$$

Now, the invariance term is:

$$s=\underset{s_{u}}{\underbrace{\frac{1}{\left| \mathcal{X}_{u} \right|}\sum_{i=1}^{\left| \mathcal{X}_{u} \right|} \left\| z_{i}-z_{2}^{'} \right\|_{2}^{2}}}+\underset{s_{l}}{\underbrace{\frac{1}{\left| \mathcal{X}_{l} \right|}\sum_{i=\left| \mathcal{X}_{u} \right|+1}^{\left| \mathcal{X}_{u} \right|+\left| \mathcal{X}_{l} \right|} \left\| z_{i}-z_{i}^{'} \right\|_{2}^{2}}}$$

Lastly, the covariance term is:

$$C\left( Z \right)=\frac{1}{\left| \mathcal{X}_{u} \right|+\left| \mathcal{X}_{l} \right|-1}\left( \underset{C_{u}}{\underbrace{\sum_{i=1}^{\left| \mathcal{X}_{u} \right|} \left( z_{i}-\bar{z} \right)\left( z_{i}-\bar{z} \right)^{T}}}+\underset{C_{l}}{\underbrace{\sum_{i=\left| \mathcal{X}_{u} \right|+1}^{\left| \mathcal{X}_{u} \right|+\left| \mathcal{X}_{l} \right|} \left( z_{i}-\bar{z} \right)\left( z_{i}-\bar{z} \right)^{T}}} \right)$$

where

$$\bar{z}=\frac{1}{\left| \mathcal{X}_{u} \right|+\left| \mathcal{X}_{l} \right|}\left( \underset{\bar{z_{u}}}{\underbrace{\sum_{i=1}^{\left| \mathcal{X}_{u} \right|} z_{i}}}+\underset{\bar{z_{l}}}{\underbrace{\sum_{i=\left| \mathcal{X}_{u} \right|+1}^{\left| \mathcal{X}_{u} \right|+\left| \mathcal{X}_{l} \right|} z_{i}}} \right)$$

Given all these loss terms, the overall loss function is now:

$$\mathcal{L}_{VICReg}=\lambda s\left( Z,Z^{'} \right)+\mu\left[ v\left( Z \right)+v\left( Z^{'} \right) \right]+\nu\left[ c\left( Z \right)+c\left( Z^{'} \right) \right]$$

where $\lambda$, $\mu$, and $\nu$ are hyperparameters to weight the effect of each loss term. Note that in the case where $v_{l,1}=\tilde{v}_{l, 2} =\emptyset,$(i.e., $\mathcal{P}_{l}=\mathcal{P}_{u}=\emptyset$), our method reduces to the default VICReg formulation.

Appendix D. Implementation Details

In this section, we provide all needed implementation details and hyperparameters for reproducing our model.

## **Appendix D.1. Pre-training**

- **Cropping:**
  - Size: 224$\times$224 pixels
  - Scale range: (0.08, 1.0)
  - Ratio range: (3/4, 4/3)
- **Encoder:** ResNet18^2^
- **Epochs:** 50
- $\boldsymbol{\eta:}{10}^{-3}$
- **Gaussian blurring:**
  - Kernel size: 23$\times$23 pixels
  - Standard deviation range: [0.1, 2.0]
- **Learning rate:** 0.45
- **Learning rate scheduler:** Cosine decay^6^
- **Learning rate warm-up epochs:** 5
- **Mini-batch size:** 256
- **Momentum:** 0.9
- **Optimizer:** LARS^7^
- **View 1 augmentations:**
  - Cropping probability: 1.0
  - Flipping probability: 0.5
  - Gaussian blurring probability: 1.0
- **View 2 augmentations:**
  - Cropping probability: 1.0
  - Flipping probability: 0.5
  - Gaussian blurring probability: 0.1
- **Weight decay:** $\boldsymbol{10}^{\boldsymbol{-6}}$
- **BYOL-specific:**
  - Projectors $g_{\theta}$ and $g_{\xi}$:
    - Number of layers: 2
    - Hidden dimension: 4096
    - Output dimension: 256
  - Predictor $q_{\theta}$:
    - Number of layers: 2
    - Hidden dimension: 4096
    - Output dimension: 256
  - $\tau$: 0.97
- **SimCLR-specific:**
  - Projector $g_{\phi}$:
    - Number of layers: 2
    - Hidden dimension: 4096
    - Output dimension: 256
  - $\tau$: 1.0
- **VICReg-specific:**
  - Expander $h_{\psi}$:
    - Number of layers: 2
    - Hidden dimension: 2048
    - Output dimension: 2048
  - $\mu$: 25
  - $\lambda$: 25
  - $\nu$: 1
  - $\epsilon$: ${10}^{-4}$

## **Appendix D.2. Linear**

- **Cropping:**
  - Size: 224$\times$224 pixels
  - Scale range: (0.08, 1.0)
  - Ratio range: (3/4, 4/3)
- **Data augmentations:**
  - Cropping probability: 1.0
  - Flipping probability: 0.5
- **Epochs:** 80
- **Learning rate:** 0.2
- **Learning rate scheduler:** Cosine decay^6^
- **Learning rate warm-up epochs:** 5
- **Mini-batch size:** 256
- **Optimizer:** SGD with Nesterov mometum^8^

## **Appendix D.3. Fully-Supervised**

- **Color jittering:**
  - Brightness factor: 0.5
  - Contrast factor: 0.5
  - Hue factor: 0.2
  - Saturation factor: 0.5
- **Data augmentations:**
  - Color jittering probability: 1.0
  - Flipping probability: 1.0
  - Rotation probability: 1.0
- **Epochs:** 40
- **Learning rate:** $\boldsymbol{10}^{\boldsymbol{-4}}$
- **Learning rate scheduler:** Decay by a factor of 0.85 each epoch
- **Mini-batch size:** 16
- **Optimizer:** Adam^9^
- **Model:** ResNet18^2^
- **Weight decay:** ${10}^{-4}$

Appendix E. Dataset Statistics

## **Appendix E.1. Celiac Disease Dataset**

In Supplementary Tables 2 and 3 we present the dataset distribution for the CD dataset at the patch- and slide-level, respectively.

| Class | Training | Development | Testing |
| --- | --- | --- | --- |
| Normal Nonspecific duodenitis | 81,428 80,090 | 4,535  9,049 | 5,966 10,010 |
| Celiac sprue | 81,560 | 9,593 | 10,038 |
| Total | 243,078 | 23,177 | 25,683 |

**Supplementary Table 2.** The Celiac disease patch-level dataset splits for the Normal, Nonspecific duodenitis, and Celiac sprue classes. The assigned label per patch is the corresponding slide-level label.

| Class | Training | Development | Testing |
| --- | --- | --- | --- |
| Normal Nonspecific duodenitis | 75 83 | 19  16 | 16 18 |
| Celiac sprue | 93 | 19 | 18 |
| Total | 251 | 54 | 52 |

**Supplementary Table 3.** The Celiac disease slide-level dataset splits for the Normal, Nonspecific duodenitis, and Celiac sprue classes.

## **Appendix E.2. Renal Cell Carcinoma Dataset**

In Supplementary Tables 4 and 5 we present the dataset distribution for the RCC dataset at the patch- and slide-level, respectively.

| Class | Training | Development | Testing |
| --- | --- | --- | --- |
| Benign | 80,165 | 1,963 | 4,372 |
| Oncocytoma | 80,903 | 1,457 | 5,470 |
| Chromophobe | 80,660 | 3,279 | 10,387 |
| Clear cell | 78,355 | 3,059 | 12,446 |
| Papillary | 83,867 | 2,259 | 9,987 |
| Total | 403,950 | 12,017 | 42,662 |

**Supplementary Table 4.** The Renal Cell Carcinoma patch-level dataset splits for the Benign, Oncocytoma, Chromophobe, Clear cell, and Papillary classes. The assigned label per patch is the corresponding slide-level label.

| Class | Training | Development | Testing |
| --- | --- | --- | --- |
| Benign | 14 | 5 | 10 |
| Oncocytoma | 14 | 3 | 10 |
| Chromophobe | 15 | 5 | 18 |
| Clear cell | 285 | 5 | 20 |
| Papillary | 55 | 5 | 20 |
| Total | 383 | 23 | 78 |

**Supplementary Table 5.** The Renal Cell Carcinoma slide-level dataset splits for the Benign, Oncocytoma, Chromophobe, Clear cell, and Papillary classes.

REFERENCES

1. Riba E, Mishkin D, Ponsa D, Rublee E, Bradski G. Kornia: an Open Source Differentiable Computer Vision Library for PyTorch. In: *Proc. IEEE/CVF Winter Conf. Appl. Comput. Vis. (WACV)*. ; 2020.

2. He K, Zhang X, Ren S, Sun J. Deep Residual Learning for Image Recognition. In: *CVPR*. ; 2015:770-778. doi:10.48550/ARXIV.1512.03385

3. Chen X, He K. Exploring Simple Siamese Representation Learning. In: *CVPR*. ; 2021:15750-15758.

4. Chen T, Kornblith S, Norouzi M, Hinton G. A Simple Framework for Contrastive Learning of Visual Representations. In: III HD, Singh A, eds. *ICML*. Vol 119. PMLR; 2020:1597-1607.

5. Bardes A, Ponce J, LeCun Y. VICReg: Variance-Invariance-Covariance Regularization for Self-Supervised Learning. In: *ICLR*. ; 2022. https://openreview.net/forum?id=xm6YD62D1Ub

6. Loshchilov I, Hutter F. SGDR: Stochastic Gradient Descent with Warm Restarts. In: *ICLR*. ; 2017.

7. You Y, Gitman I, Ginsburg B. Large Batch Training of Convolutional Networks. Published online 2017. doi:10.48550/ARXIV.1708.03888

8. Sutskever I, Martens J, Dahl G, Hinton G. On the importance of initialization and momentum in deep learning. In: Dasgupta S, McAllester D, eds. *ICML*. Vol 28. PMLR; 2013:1139-1147.

9. Kingma DP, Ba J. Adam: A Method for Stochastic Optimization. In: Bengio Y, LeCun Y, eds. *ICLR*. ; 2015. http://arxiv.org/abs/1412.6980
